# *N*′-[(*E*)-(5-Methyl­furan-2-yl)methyl­idene]formohydrazide

**DOI:** 10.1107/S1600536809037064

**Published:** 2009-09-19

**Authors:** Zahid Shafiq, Muhammad Yaqub, M. Nawaz Tahir, Mian Hasnain Nawaz, M. Saeed Iqbal

**Affiliations:** a Department of Chemistry, Bahauddin Zakariya University, Multan 60800, Pakistan; b Department of Physics, University of Sargodha, Sargodha, Pakistan; c Department of Chemistry, Government College University, Lahore, Pakistan

## Abstract

The title compound, C_7_H_8_N_2_O_2_, is almost planar (r.m.s. deviation for non-H atoms = 0.029 Å). In the crystal, inversion dimers linked by pairs of N—H⋯O hydrogen bonds generate an *R*_2_^2^(8) ring motif.

## Related literature

For related structures, see: Shafiq *et al.* (2009 ▶); Bai & Jing (2007 ▶); Yao & Jing (2007 ▶). For graph-set notation, see: Bernstein *et al.* (1995 ▶).
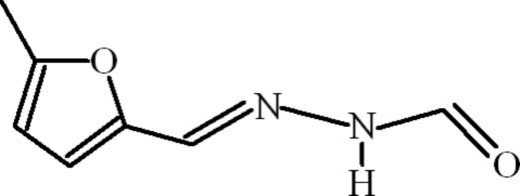


## Experimental

### 

#### Crystal data

C_7_H_8_N_2_O_2_
*M* *_r_* = 152.15
Orthorhombic, 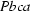

*a* = 10.6433 (14) Å
*b* = 6.7762 (8) Å
*c* = 21.129 (3) Å
*V* = 1523.9 (3) Å^3^
*Z* = 8
Mo *K*α radiation
μ = 0.10 mm^−1^
*T* = 296 K
0.25 × 0.15 × 0.13 mm

#### Data collection

Bruker Kappa APEXII CCD diffractometer
Absorption correction: multi-scan (*SADABS*; Bruker, 2005 ▶) *T*_min_ = 0.985, *T*_max_ = 0.988
7485 measured reflections
1403 independent reflections
655 reflections with *I* > 2σ(*I*)
*R*_int_ = 0.072

#### Refinement

*R*[*F* ^2^ > 2σ(*F* ^2^)] = 0.044
*wR*(*F* ^2^) = 0.108
*S* = 1.00
1403 reflections
101 parameters
H-atom parameters constrained
Δρ_max_ = 0.12 e Å^−3^
Δρ_min_ = −0.16 e Å^−3^

### 

Data collection: *APEX2* (Bruker, 2007 ▶); cell refinement: *SAINT* (Bruker, 2007 ▶); data reduction: *SAINT*; program(s) used to solve structure: *SHELXS97* (Sheldrick, 2008 ▶); program(s) used to refine structure: *SHELXL97* (Sheldrick, 2008 ▶); molecular graphics: *ORTEP-3 for Windows* (Farrugia, 1997 ▶) and *PLATON* (Spek, 2009 ▶); software used to prepare material for publication: *WinGX* (Farrugia, 1999 ▶) and *PLATON*.

## Supplementary Material

Crystal structure: contains datablocks global, I. DOI: 10.1107/S1600536809037064/hb5099sup1.cif

Structure factors: contains datablocks I. DOI: 10.1107/S1600536809037064/hb5099Isup2.hkl

Additional supplementary materials:  crystallographic information; 3D view; checkCIF report

## Figures and Tables

**Table 1:** Hydrogen-bond geometry (Å, °)

*D*—H⋯*A*	*D*—H	H⋯*A*	*D*⋯*A*	*D*—H⋯*A*
N2—H2*A*⋯O2^i^	0.86	2.00	2.848 (3)	169

Symmetry code: (i) 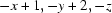.

**Fig. 1.**
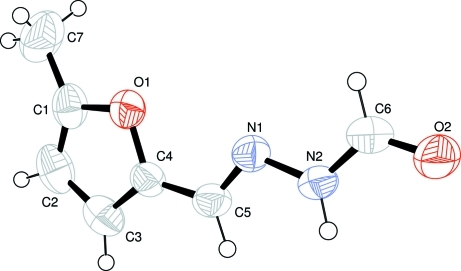
View of (I) with displacement ellipsoids drawn at the 50% probability level. H-atoms are shown by small circles of arbitrary radius.

**Fig. 2.**
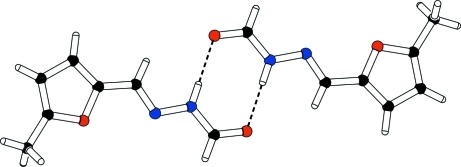
The partial packing if (I) which shows that molecules are dimerized and form ring motifs.

## supplementary crystallographic information

### Comment

In continuation of our studies of different derivatives of formohydrazide (Shafiq *et al.*, 2009), the title compound (I, Fig. 1), has been prepared and being reported. The metal complexes of (I) has been prepared with transition metals and their various studies are in progress.

The crystal structures of (II) (*E*)-4-bromo-*N*'-((5-methylfuran-2-yl)methylene)benzohydrazide (Bai & Jing, 2007), (III) (*E*)-*N*'-((5-methylfuran-2-yl)methylene)furan-2-carbohydrazide (Yao & Jing, 2007) have been reported which contain the 5-methylfuran-2-yl moiety as present in (I).  The title compound consists of dimers due to intermolecular H-bonding of type N–H···O (Table 1, Fig. 2) forming *R*_2_^2^(8) (Bernstein *et al.*, 1995) ring motif. Similar bonding also exist in *N*'-[(1*E*)-1-(4-Chlorophenyl)ethylidene]formohydrazide (Shafiq *et al.*, 2009). The overall molecule of (I) is planar with an r.m.s. deviation of 0.0285 Å.

### Experimental

To a hot stirred solution of formohydrazide (1.0 g, 0.017 mol) in ethanol (10 ml) was added 5-methylfurfural (1.65 ml, 0.017 mol). The resultant mixture was then heated under reflux for 4 h and monitored through TLC. After completion of reaction, the mixture was cooled to room temperature. The crude solid was collected by suction filtration. The precipitates were washed with hot ethanol, filtered and dried. Brown needles of (I) were obtained by recrystalization from (1:1 v/v) methanol:1,4-dioxan.

### Refinement

The H-atoms were positioned geometrically with N—H = 0.86, C—H = 0.93 and 0.96 Å for aryl and methyl H atoms, respectively and constrained to ride on their parent atoms, with *U*_iso_(H) = xUeq(C,*N*), where *x* = 1.5 for methyl H and *x* = 1.2 for all other H atoms.

### Crystal data

**Table:**

C_7_H_8_N_2_O_2_	*F*(000) = 640
*M**_r_* = 152.15	*D*_x_ = 1.326 Mg m^−^^3^
Orthorhombic, *P**b**c**a*	Mo *K*α radiation, λ = 0.71073 Å
Hall symbol: -P 2ac 2ab	Cell parameters from 1864 reflections
*a* = 10.6433 (14) Å	θ = 2.7–25.5°
*b* = 6.7762 (8) Å	µ = 0.10 mm^−^^1^
*c* = 21.129 (3) Å	*T* = 296 K
*V* = 1523.9 (3) Å^3^	Cut needle, brown
*Z* = 8	0.25 × 0.15 × 0.13 mm

### Data collection

**Table:**

Bruker Kappa APEXII CCD diffractometer	1403 independent reflections
Radiation source: fine-focus sealed tube	655 reflections with *I* > 2σ(*I*)
graphite	*R*_int_ = 0.072
Detector resolution: 7.80 pixels mm^-1^	θ_max_ = 25.5°, θ_min_ = 2.7°
ω scans	*h* = −12→12
Absorption correction: multi-scan (*SADABS*; Bruker, 2005)	*k* = −5→8
*T*_min_ = 0.985, *T*_max_ = 0.988	*l* = −23→25
7485 measured reflections	

### Refinement

**Table:**

Refinement on *F*^2^	Primary atom site location: structure-invariant direct methods
Least-squares matrix: full	Secondary atom site location: difference Fourier map
*R*[*F*^2^ > 2σ(*F*^2^)] = 0.044	Hydrogen site location: inferred from neighbouring sites
*wR*(*F*^2^) = 0.108	H-atom parameters constrained
*S* = 1.00	*w* = 1/[σ^2^(*F*_o_^2^) + (0.0376*P*)^2^] where *P* = (*F*_o_^2^ + 2*F*_c_^2^)/3
1403 reflections	(Δ/σ)_max_ < 0.001
101 parameters	Δρ_max_ = 0.12 e Å^−^^3^
0 restraints	Δρ_min_ = −0.16 e Å^−^^3^

### Special details

**Table:**

Geometry. Bond distances, angles *etc*. have been calculated using the rounded fractional coordinates. All su's are estimated from the variances of the (full) variance-covariance matrix. The cell e.s.d.'s are taken into account in the estimation of distances, angles and torsion angles
Refinement. Refinement of *F*^2^ against ALL reflections. The weighted *R*-factor *wR* and goodness of fit *S* are based on *F*^2^, conventional *R*-factors *R* are based on *F*, with *F* set to zero for negative *F*^2^. The threshold expression of *F*^2^ > σ(*F*^2^) is used only for calculating *R*-factors(gt) *etc*. and is not relevant to the choice of reflections for refinement. *R*-factors based on *F*^2^ are statistically about twice as large as those based on *F*, and *R*- factors based on ALL data will be even larger.

### Fractional atomic coordinates and isotropic or equivalent isotropic displacement parameters (Å^2^)

**Table:**

	*x*	*y*	*z*	*U*_iso_*/*U*_eq_	
O1	0.56771 (16)	0.2958 (2)	0.15544 (8)	0.0550 (7)	
O2	0.66434 (16)	1.0759 (3)	0.01153 (9)	0.0665 (8)	
N1	0.5699 (2)	0.6414 (3)	0.08604 (10)	0.0486 (8)	
N2	0.56384 (19)	0.8059 (3)	0.04784 (10)	0.0497 (8)	
C1	0.5376 (3)	0.1184 (4)	0.18332 (14)	0.0602 (11)	
C2	0.4218 (3)	0.0664 (4)	0.16579 (15)	0.0717 (14)	
C3	0.3762 (3)	0.2130 (4)	0.12461 (15)	0.0641 (11)	
C4	0.4667 (2)	0.3504 (4)	0.11942 (12)	0.0481 (10)	
C5	0.4735 (2)	0.5290 (4)	0.08350 (13)	0.0501 (10)	
C6	0.6618 (2)	0.9277 (4)	0.04483 (14)	0.0542 (11)	
C7	0.6371 (3)	0.0296 (4)	0.22318 (15)	0.0950 (16)	
H2	0.37892	−0.04640	0.17850	0.0858*	
H2A	0.49724	0.82967	0.02609	0.0596*	
H3	0.29823	0.21455	0.10475	0.0770*	
H5	0.40630	0.56423	0.05772	0.0599*	
H6	0.73203	0.89887	0.06930	0.0651*	
H7A	0.61090	−0.09910	0.23690	0.1421*	
H7B	0.71316	0.01834	0.19904	0.1421*	
H7C	0.65168	0.11181	0.25943	0.1421*	

### Atomic displacement parameters (Å^2^)

**Table:**

	*U*^11^	*U*^22^	*U*^33^	*U*^12^	*U*^13^	*U*^23^
O1	0.0546 (12)	0.0537 (12)	0.0566 (13)	−0.0005 (9)	0.0025 (11)	0.0096 (10)
O2	0.0620 (14)	0.0531 (12)	0.0845 (17)	−0.0070 (9)	−0.0148 (11)	0.0163 (11)
N1	0.0461 (14)	0.0486 (13)	0.0510 (16)	0.0081 (12)	0.0010 (12)	0.0037 (12)
N2	0.0408 (13)	0.0512 (13)	0.0570 (16)	0.0048 (12)	−0.0071 (12)	0.0094 (12)
C1	0.074 (2)	0.0447 (18)	0.062 (2)	0.0016 (16)	0.0148 (19)	0.0060 (16)
C2	0.080 (2)	0.054 (2)	0.081 (3)	−0.0146 (18)	0.025 (2)	−0.0025 (17)
C3	0.0533 (19)	0.068 (2)	0.071 (2)	−0.0095 (17)	0.0073 (17)	−0.0096 (18)
C4	0.0415 (16)	0.0546 (19)	0.0483 (19)	0.0013 (15)	0.0047 (14)	−0.0011 (15)
C5	0.0416 (16)	0.0567 (18)	0.052 (2)	0.0088 (14)	−0.0007 (14)	0.0003 (15)
C6	0.0454 (18)	0.0562 (18)	0.061 (2)	0.0042 (15)	−0.0088 (16)	−0.0033 (17)
C7	0.116 (3)	0.081 (2)	0.088 (3)	0.018 (2)	−0.004 (2)	0.031 (2)

### Geometric parameters (Å, °)

**Table:**

O1—C1	1.377 (3)	C3—C4	1.344 (4)
O1—C4	1.368 (3)	C4—C5	1.430 (4)
O2—C6	1.227 (3)	C2—H2	0.9300
N1—N2	1.378 (3)	C3—H3	0.9300
N1—C5	1.279 (3)	C5—H5	0.9300
N2—C6	1.331 (3)	C6—H6	0.9300
N2—H2A	0.8600	C7—H7A	0.9600
C1—C2	1.334 (4)	C7—H7B	0.9600
C1—C7	1.481 (4)	C7—H7C	0.9600
C2—C3	1.407 (4)		
			
O1···N1	2.763 (3)	C6···N1^i^	3.318 (3)
O2···N1^i^	3.268 (3)	C6···C1^iv^	3.461 (4)
O2···N2^ii^	2.848 (3)	C6···O2^iii^	3.099 (3)
O2···C6^i^	3.099 (3)	C1···H7A^vii^	3.0000
O1···H6^iii^	2.8900	C2···H7A^vii^	3.0800
O2···H2A^ii^	2.0000	C6···H2A^ii^	2.8000
O2···H6^i^	2.7400	H2A···H5	2.1500
N1···O1	2.763 (3)	H2A···O2^ii^	2.0000
N1···O2^iii^	3.268 (3)	H2A···C6^ii^	2.8000
N1···C6^iii^	3.318 (3)	H2A···H2A^ii^	2.5600
N2···C2^iv^	3.408 (4)	H5···H2A	2.1500
N2···O2^ii^	2.848 (3)	H6···O1^i^	2.8900
N1···H6^iii^	2.7000	H6···O2^iii^	2.7400
C1···C6^v^	3.461 (4)	H6···N1^i^	2.7000
C2···N2^v^	3.408 (4)	H7A···C1^viii^	3.0000
C5···C5^vi^	3.595 (4)	H7A···C2^viii^	3.0800
			
C1—O1—C4	106.9 (2)	C1—C2—H2	126.00
N2—N1—C5	114.8 (2)	C3—C2—H2	126.00
N1—N2—C6	119.5 (2)	C2—C3—H3	127.00
N1—N2—H2A	120.00	C4—C3—H3	127.00
C6—N2—H2A	120.00	N1—C5—H5	119.00
C2—C1—C7	135.3 (3)	C4—C5—H5	119.00
O1—C1—C2	109.1 (2)	O2—C6—H6	118.00
O1—C1—C7	115.6 (2)	N2—C6—H6	118.00
C1—C2—C3	107.7 (3)	C1—C7—H7A	109.00
C2—C3—C4	107.0 (3)	C1—C7—H7B	109.00
O1—C4—C5	119.0 (2)	C1—C7—H7C	109.00
O1—C4—C3	109.3 (2)	H7A—C7—H7B	109.00
C3—C4—C5	131.7 (2)	H7A—C7—H7C	109.00
N1—C5—C4	121.5 (2)	H7B—C7—H7C	110.00
O2—C6—N2	123.5 (2)		
			
C4—O1—C1—C2	−0.7 (3)	O1—C1—C2—C3	0.9 (3)
C4—O1—C1—C7	178.2 (2)	C7—C1—C2—C3	−177.7 (3)
C1—O1—C4—C3	0.2 (3)	C1—C2—C3—C4	−0.7 (4)
C1—O1—C4—C5	−178.5 (2)	C2—C3—C4—O1	0.3 (3)
C5—N1—N2—C6	−177.0 (2)	C2—C3—C4—C5	178.8 (3)
N2—N1—C5—C4	178.7 (2)	O1—C4—C5—N1	−2.4 (4)
N1—N2—C6—O2	179.2 (2)	C3—C4—C5—N1	179.2 (3)

Symmetry codes: (i) −*x*+3/2, *y*+1/2, *z*; (ii) −*x*+1, −*y*+2, −*z*; (iii) −*x*+3/2, *y*−1/2, *z*; (iv) *x*, *y*+1, *z*; (v) *x*, *y*−1, *z*; (vi) −*x*+1, −*y*+1, −*z*; (vii) −*x*+1, *y*+1/2, −*z*+1/2; (viii) −*x*+1, *y*−1/2, −*z*+1/2.

### Hydrogen-bond geometry (Å, °)

**Table:**

*D*—H···*A*	*D*—H	H···*A*	*D*···*A*	*D*—H···*A*
N2—H2A···O2^ii^	0.86	2.00	2.848 (3)	169

Symmetry codes: (ii) −*x*+1, −*y*+2, −*z*.
